# Evaluation of the iPhone with an acrylic sleeve versus the Scoliometer for rib hump measurement in scoliosis

**DOI:** 10.1186/1748-7161-7-14

**Published:** 2012-07-30

**Authors:** Maree T Izatt, Gary R Bateman, Clayton J Adam

**Affiliations:** 1 Paediatric Spine Research Group, Queensland University of Technology and Mater Health Services, Level 2, Mater Children’s Hospital, Raymond Terrace, South Brisbane, Queensland, 4101, Australia; 2 GB Orthopaedics Australia, 39 Annerley Road, South Brisbane, Queensland, 4101, Australia

**Keywords:** Scoliosis, Rib hump, Rib hump measurement, Scoliometer, iPhone, Measurement variability, Smartphone, Inter-observer variability, Intra-observer variability

## Abstract

**Background:**

Vertebral rotation found in structural scoliosis contributes to trunkal asymmetry which is commonly measured with a simple Scoliometer device on a patient's thorax in the forward flexed position. The new generation of mobile 'smartphones' have an integrated accelerometer, making accurate angle measurement possible, which provides a potentially useful clinical tool for assessing rib hump deformity. This study aimed to compare rib hump angle measurements performed using a Smartphone and traditional Scoliometer on a set of plaster torsos representing the range of torsional deformities seen in clinical practice.

**Methods:**

Nine observers measured the rib hump found on eight plaster torsos moulded from scoliosis patients with both a Scoliometer and an Apple iPhone on separate occasions. Each observer repeated the measurements at least a week after the original measurements, and were blinded to previous results. Intra-observer reliability and inter-observer reliability were analysed using the method of Bland and Altman and 95% confidence intervals were calculated. The Intra-Class Correlation Coefficients (ICC) were calculated for repeated measurements of each of the eight plaster torso moulds by the nine observers.

**Results:**

Mean absolute difference between pairs of iPhone/Scoliometer measurements was 2.1 degrees, with a small (1 degrees) bias toward higher rib hump angles with the iPhone. 95% confidence intervals for intra-observer variability were +/- 1.8 degrees (Scoliometer) and +/- 3.2 degrees (iPhone). 95% confidence intervals for inter-observer variability were +/- 4.9 degrees (iPhone) and +/- 3.8 degrees (Scoliometer). The measurement errors and confidence intervals found were similar to or better than the range of previously published thoracic rib hump measurement studies.

**Conclusions:**

The iPhone is a clinically equivalent rib hump measurement tool to the Scoliometer in spinal deformity patients. The novel use of plaster torsos as rib hump models avoids the variables of patient fatigue and discomfort, inconsistent positioning and deformity progression using human subjects in a single or multiple measurement sessions.

## Background

**Figure 1:**
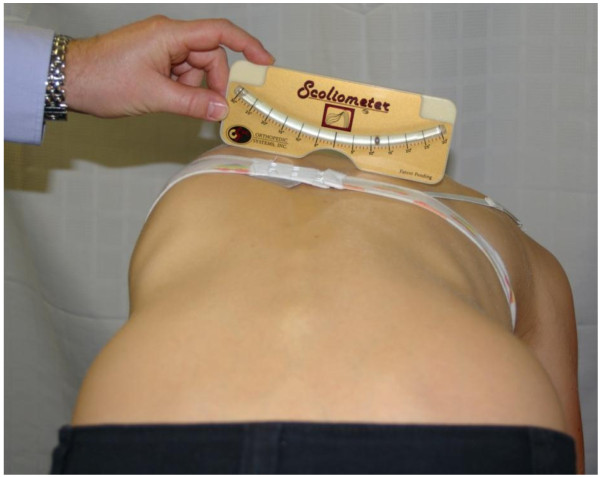
Measurement of the rib hump of a scoliosis patient in the forward bending position using a Scoliometer where the examiner is looking for the highest reading detected in the thoracic spine.

Vertebral rotation is a key distinguishing feature in the diagnosis of structural scoliosis. Axial vertebral rotation combined with rotary distortion within the spinal vertebrae contribute to the overall trunkal asymmetry found in the scoliosis patient which is most easily visible when the patient is standing in the forward flexed position, known as the Adams forward bending test [1]. In the forward flexed position, the ribcage is elevated on the side of the scoliotic curve convexity and depressed on the side of the concavity. Measurement of the resulting angle of thorax rotation or rib hump on patients with scoliosis is routine practice in spinal clinics and school screening programs worldwide to easily monitor the presence and progression of the vertebral rotation (Figure 1). A simple inclinometer device, called a Scoliometer (National Scoliosis Foundation, Watertown, MA), was introduced in 1984 by Bunnell [2] with the aim of reducing the number of radiographs taken as a result of scoliosis screening programs. Used in conjunction with Cobb angle measurements on plain or digital radiographs, the Scoliometer continues to be used to the present day.

Previous studies [3-7] have found the Scoliometer to have adequate or high inter-rater and intra-rater reliability coefficients indicating good measurement reproducibility. The most recent of these by Bonagamba et al. [7], found the highest intra-rater and inter-rater reliability of the Scoliometer to date, as a result of minimising the major sources of measurement variability in earlier studies. It was suggested the process of patient positioning, vertebral level palpation, patient discomfort and fatigue with being positioned and measured multiple times, and repeat measurements occurring weeks apart, all contributed to Scoliometer measurement variability in prior studies.

The iPhone (Apple Inc, Cupertino, USA) is one of a recent generation of mobile phones which incorporate a MEMS (micro-electro-mechanical-system) accelerometer, which can accurately sense acceleration and inclination. The availability of various software applications for the iPhone which read and display the accelerometer signal allow it to be used potentially in a wide range of clinical applications to replace for example; the goniometer to measure peripheral joint ranges of motion, the protractor to measure Cobb angles [8], and in this study the Scoliometer for rib hump assessment in spinal deformity patients. The aim of this study was to quantify the measurement performance of the iPhone compared to the standard Scoliometer for the assessment of vertebral rotation in structural scoliosis.

This study also aimed to further minimise the major sources of measurement variability by using a set of plaster rib hump models of actual scoliosis patients retrieved from a specialist spinal orthotist who produces full body plaster torsos in the process of manufacturing custom made scoliosis braces. As a result, the current study eliminated patient inconvenience, the variability of patient positioning and posture, subject discomfort and fatigue, and the possibility of deformity progression between measurement sessions by multiple observers or the same observer. By minimising the known variables that occur when performing multiple measurements, this study aimed to better compare the measurement performance of the devices (iPhone and Scoliometer) rather than the combination of the measurement method and the devices.

## Methods

### Study specimens

**Figure 2:**
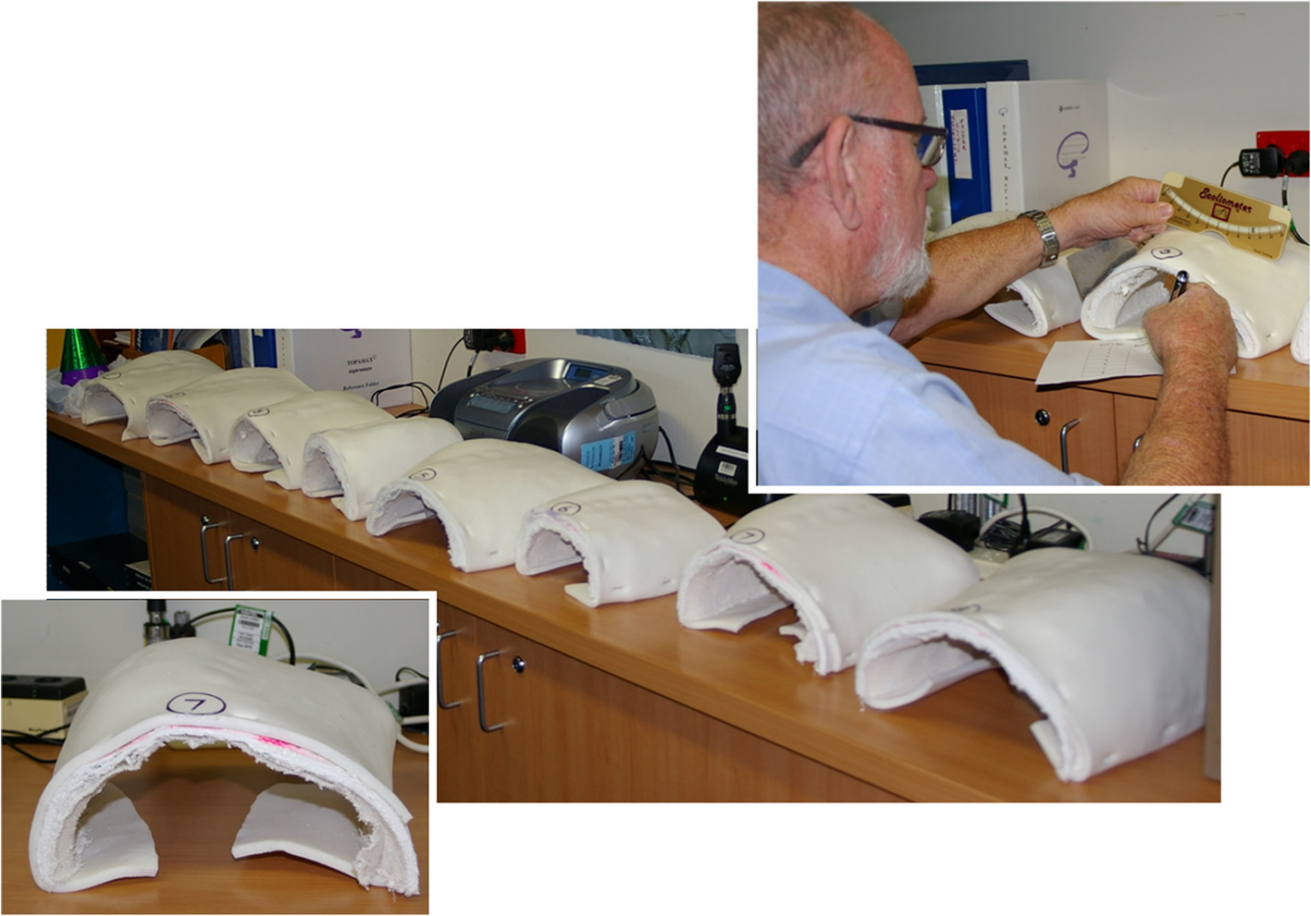
Plaster moulds of eight scoliosis patient’s rib humps arranged in random order on a standard height bench in preparation for measurement by each observer with either the Scoliometer or iPhone.

Eight plaster torsos were retrieved when they were no longer required from a spine brace manufacturer in Brisbane, Australia. The solid plaster torsos had been produced by an experienced spinal Orthotist during the process of having a custom-fit spinal brace made to control a progressive scoliosis deformity. The Orthotist made plaster moulds of the rib hump regions and overlaid a 7 mm foam layer to mimic the skin surface. As well as significantly reducing the weight of the solid plaster torso, this also ensured the rib hump models were easily portable and would consistently sit flat on the table surface each week they were measured (see below). Finally, the plaster rib hump moulds were numbered 1 – 8. The collection of plaster rib hump moulds represented a range of ribcage distortions and severities that would remain static during multiple measurement sessions (Figure 2).

### Rib hump measurements

The plaster moulds were placed on a standard height bench in random order on four occasions, one week apart. Observers measured the rib humps with either the iPhone or the Scoliometer each week until all observers had measured the humps twice with each device. For all measurements, the observers were unaware of all previous measurements. The observers were free to select the location on the plaster model of the most severe rib hump angle, as would be the case when measuring the rib hump angle on the thorax of a spinal deformity patient in the forward flexed position. The observers were seated during angle measurements to ensure the moulds were consistently positioned at around eye level.

**Figure 3:**
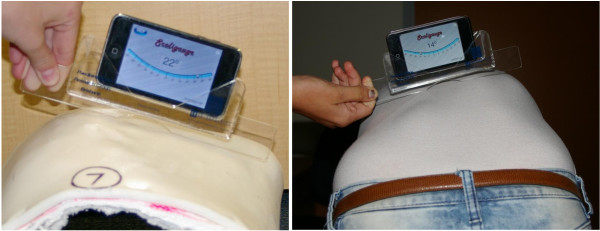
Measurement of the rib hump on a (a) plaster model and a (b) scoliosis patient, using the iPhone and Scolioguage software in combination with the acrylic sleeve to reflect the dimensions of the traditional Scoliometer.

**Figure 4:**
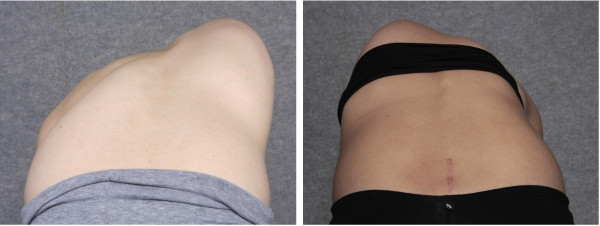
Photographs of two scoliosis patients in the forward flexed position where use of a smartphone in isolation (without the acrylic sleeve) to measure the rib hump would result in failure to cover the full extent of the trunk rotational deformity.

Nine observers of varying skill level (four experienced spinal orthopaedic surgeons, a specialist physiotherapist, an experienced spinal orthotist, two training grade registrars and an inexperienced physiotherapist) measured the rib hump angles of the eight plaster torsos using the Scoliometer and the iPhone. The iPhone rib hump measurements were performed using the Scoliguage application software (Ockendon Partners Ltd, UK, http://www.ockendon.net), which was downloaded from the Apple iTunes store. When using the iPhone to measure rib humps, all observers used an acrylic sleeve designed to accommodate any inclinometer equipped device, and to approximate the length and shape of the standard Scoliometer (Figure 3). The additional length provided by the drop-in sleeve, ensured full coverage of the rib hump deformity which may be underestimated in severe deformity cases should a smartphone be used in isolation (see examples in Figure 4). The observers were free to perform the measurements of the eight torsos in any order during the four week period but could only perform one set of measurements each week to avoid the recall of any prior measurements. Each set of measurements were recorded on separate data entry sheets and placed through a slot into a sealed box for the duration of the study. Inter and intra-observer variability associated with the two measurement techniques was assessed as described below.

### Statistical analysis

The two rib hump measurement devices were compared using the approach described by Bland and Altman [9-11] to assess agreement between subsequent measures by the same examiner (intra-observer reliability) and agreement between the measurements made by different examiners (inter-observer reliability). Intra-observer variability was assessed by analysing the absolute difference between successive rib hump angle (*α*) measurements by the same observer using the same measurement tool,

$$\Delta \alpha =\left|\alpha_{n}-\alpha_{n+1}\right|$$

where *n* and *n* + 1 are successive measurements. 95% confidence intervals for intra-observer variability were calculated as (1.96×*SD*_*intra*_) [9,10] where *SD*_*intra*_ is the standard deviation of the intra-observer differences *Δα*.

The inter-observer variability (standard deviation of the difference between measurements by two different observers) was calculated as √2×*SD*_*inter*_ for a single measurement per observer, where *SD*_*inter*_ is the standard deviation of the inter-observer differences [9]. The 95% confidence intervals for inter-observer variability were calculated using 2.37×*SD* (*t*-distribution with 7 *dof*) [9].

In order to allow comparison with previous rib hump measurement variability studies, we also calculated the Intra-Class Correlation Coefficient (ICC) for repeated measurements of each of the eight plaster torso moulds by the nine observers. Both absolute agreement and consistency measures were assessed using a two-way model with SPSS (v 8.0 for Windows, SPSS Inc., Chicago, IL). Note that the absolute agreement measure is a more stringent ICC definition (ICC = 1 requires perfect agreement between all observers).

## Results

Assessment of the rib hump measurements using both devices, on eight plaster torsos by nine observers, with two assessments by each observer, gave a total of 280 out of a possible 288 measurements for analysis. One training Registrar failed to perform a second set of Scoliometer measurements. The overall mean rib hump angle for the group of plaster moulds was 16° ± 5.8 (range 6–30) representing the range of torsional deformities seen in clinical practice.

### iPhone vs scoliometer comparison

**Figure 5:**
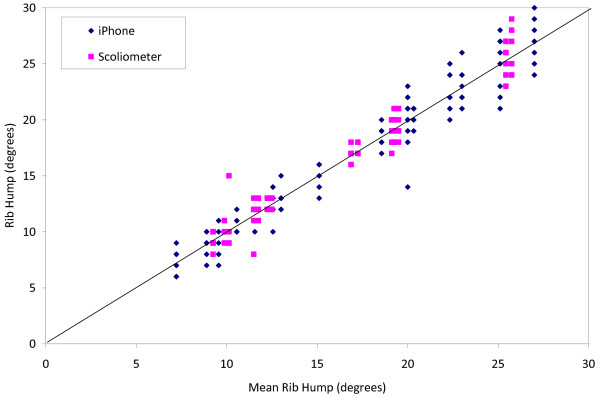
Data points for all the iPhone and Scoliometer measurements plotted versus the mean rib hump angle for each plaster torso.

**Figure 6:**
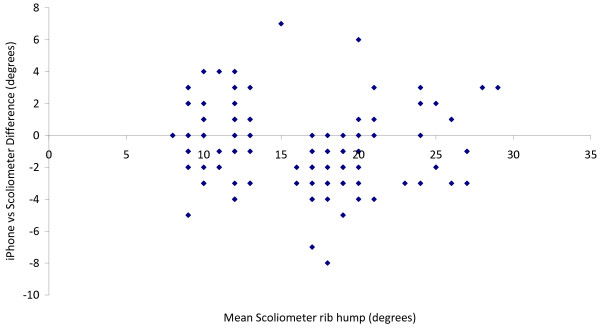
Scatter plot showing the signed measurement difference between pairs of iPhone/Scoliometer measurements performed by the same observer on the same rib hump model, plotted versus the mean Scoliometer rib hump angle.

Figure 5 shows all data points for both the iPhone and Scoliometer measurements plotted versus the mean rib hump angle for each plaster torso. Figure 6 shows a graph of signed measurement difference between pairs of iPhone/Scoliometer measurements for the same plaster torso mould, versus mean rib hump angle. The mean absolute difference between pairs of iPhone and Scoliometer measurements was 2.1° ± 1.6 (range 0–8), and the mean signed difference was −0.9° (range −8 to +7), suggesting that there is a small measurement bias of 1° toward higher rib hump angles with the iPhone. The 95% confidence interval for differences between iPhone and Scoliometer measurements on the same plaster rib hump mould was 1.96×*SD* = ±3.12°.

### Intra-observer variability

**Figure 7:**
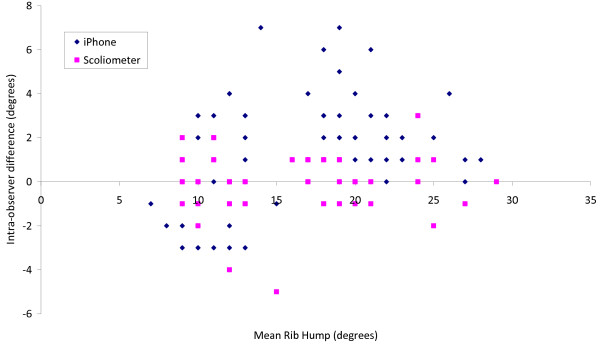
Scatter plot of intra-observer difference between successive measurements (at least one week apart) by the same observer on the same rib hump model using the same measuring tool, plotted versus mean rib hump angle for each plaster model.

Figure 7 shows the difference between pairs of successive measurements by the same observer for both the iPhone and Scoliometer, plotted versus mean rib hump angle. The mean absolute intra-observer difference was 0.9° ± 0.9 (range 0 to 5, 95% *CI* = 1.8°) for the Scoliometer, and 2.2° ± 1.6 (range 0 to 7, 95% *CI* = 3.2°) for the iPhone.

### Inter-observer variability

Based on a single reading by each observer, the *SD* of a rib hump angle measurement was 1.5° for the iPhone and 1.1° for the Scoliometer. The inter-observer error (standard deviation of the difference between measurements by two different observers) is therefore √2×*SD* = 2.1° for the iPhone and 1.6° for the Scoliometer [9]. The 95% confidence intervals for inter-observer error were ± 4.9° and ±3.8° for the iPhone and Scoliometer respectively, calculated using 2.365×*SD* (*t*-distribution with 7 *dof*).

### Intra-class correlation coefficients

For iPhone rib hump measurements, the ICC was 0.924 using an absolute agreement definition, and 0.939 using a consistency definition. For Scoliometer rib hump measurements, the ICC was 0.947 using an absolute agreement definition, and 0.950 using a consistency definition.

## Discussion

The increasing popularity of mobile phones and hand held tablets incorporating micro-electromechanical system (MEMS) accelerometers has provided a new technology for accurate angle measurements. The ubiquitous nature of these devices and the ready availability of diverse software applications mean they may have a significant impact on efficiency and convenience in school screening programs and spinal clinics for assessment and diagnosis of spinal deformities. In the medical setting, software applications are available to measure Cobb angles and peripheral joint angles, display computed tomography and magnetic resonance imaging and provide alerts regarding clinical pathology results of individual patients direct to the treating doctor. These mobile technologies offer a convenient tool for the physician; however this necessitates scientific studies to ensure that measurements reported by the smartphone can be relied upon with respect to clinical management decisions for patients. In the current study, we present a novel methodology using static rib hump moulds fabricated from scoliosis patients and use this technique to assess the measurement performance of the iPhone compared to the Scoliometer. The rib humps of the plaster models ranged from 6 to 30° which represented a large range of trunkal asymmetries with the aim of being representative of those which would be encountered in clinical practice. As with the Bunnell Scoliometer, the iPhone together with the Scoliguage software application is a simple, inexpensive and portable method of measuring rib hump progression and a practical way to decrease exposure to radiation from repeated radiographs [3,4,12].

When the Spinal Orthopaedic Surgeons at our centre began trialling the iPhone to measure the rib hump of spinal deformity patients, it became clear that the iPhone alone, was for some patients, of inadequate length. For patients with more severe and/or angular rib hump deformities, the length of a mobile phone was unable to cover the full expanse of the ribcage rotational deformity. As a result the rib hump could be underestimated for these cases. The spinal surgeons were of the opinion that additional length was required to ensure measurement accuracy for all rib hump severities which lead to the development of the smartphone acrylic sleeve. The sleeve provides the required additional length and includes the central notch on the inferior edge of the drop-in device to mirror the shape of the traditional Scoliometer which is used to facilitate the placement of the device over the central spinous processes. Due to the larger size of the more recently available hand held tablets (iPad and similar devices), these devices are useful to measure rib hump angles in isolation but were not evaluated as part of the current study.

Although the correlation between trunkal asymmetry and vertebral column deformity is beyond the scope of this paper, it is important to recognise that when considering the correlation between trunk asymmetry measures and spinal deformity, previous work by Grivas *et al.*[13], found that in children aged 7–13 years the concordance between trunk and spinal deformity was weak but became stronger for children aged 14–18 years. It should also be noted that trunkal asymmetry measurements alone are not sufficient for determining a definitive patient diagnosis and management plan [3].

In this study, the mean difference between pairs of iPhone and Scoliometer measurements was small, with a mean absolute difference of just over 2°, with a small bias of 1° toward higher rib hump angles with the iPhone and a 95% confidence interval of just over 3°. All of these figures are less than the minimum 5° difference which is widely accepted as signifying a clinically significant change in rib hump deformity. Therefore, we conclude that the iPhone is a clinically equivalent measuring tool to the traditional Scoliometer.

Furthermore, the inter- and intra-observer measurement variability using the iPhone were found to be similar to that of the Scoliometer in the current study. As with nearly all previous studies, the 95% confidence intervals for inter-observer variability were higher than those for intra-observer variability, for both the iPhone and the Scoliometer. Carman *et al.*[14] note that the intra-observer variability is a more clinically relevant parameter than the inter-observer variability because intra-observer differences can lead to misdiagnosis of rib hump progression, thus influencing clinical treatment decisions. However we note that inter-observer variability may be equally important in large public spinal clinics where different clinicians are likely to assess patients on subsequent clinic review visits. Furthermore, the Intra-class Correlation Coefficients reported in this study (ICC = 0.92-0.95) compare favourably with ICCs reported (0.81-0.95) in previous Scoliometer studies [4-7]. This is to be expected since our use of plaster rib hump moulds eliminates the variability that is due to patient posture, patient fatigue and deformity progression which may all have contributed to the measurement variability results in previous studies. We note that using the iPhone in the clinical setting to measure trunkal asymmetry is subject to patient-positioning variability, and this variability is an unavoidable clinical factor which will occur regardless of the chosen measurement device used.

## Conclusions

Clinical judgements as a result of iPhone rib hump measurements can be made with confidence based on readings taken from the iPhone when combined with the acrylic sleeve.

The inter- and intra-observer measurement variability using the iPhone were found to be similar to that of the Scoliometer.

The novel use of plaster torsos as rib hump models avoids the variables of patient fatigue and discomfort, inconsistent positioning and deformity progression using human subjects in a single or multiple measurement sessions.

## Competing interests

MTI and CJA declare they have no competing interests. GB has patent pending on the acrylic sleeve used in this study.

## Authors’ contributions

MTI participated in the conception, design and coordination of the study, collated the data, performed the rib hump measurements and data analysis, drafted and revised the manuscript. CJA participated in the conception and design of the study, data analysis, drafting and revising of the manuscript. GB designed and provided the plaster models and the acrylic smartphone sleeve, performed rib hump measurements and contributed to the drafting and review of the manuscript. All authors read and approved the final manuscript.

## Sources of support

No financial support was received for this study. None of the authors have any commercial relationship with Apple or with the producers of the Scoliguage software application mentioned in this article. GB Orthopaedics Australia (author GB) designed and provided the acrylic sleeve used in this study.
